# Effect of Short Tandem Target Mimic miR-5110 on Melanogenesis in Melanocytes of Alpaca (*Vicugna pacos*)

**DOI:** 10.3390/cimb48010072

**Published:** 2026-01-10

**Authors:** Shanshan Yang, Dingxing Jiao, Xuqi Wang, Yangyang Yan, Tao Song, Lili Wang, Ping Rui, Zengjun Ma, Fengsai Li

**Affiliations:** 1 College of Animal Science and Technology, Hebei Normal University of Science and Technology, Qinhuangdao 066000, China; shanshan0321@163.com (S.Y.); xiaowu12_3@126.com (D.J.); wangxuqi1244@163.com (X.W.); 18033571508@163.com (Y.Y.); songtaoer@126.com (T.S.); iamwanglili100@163.com (L.W.); rp1969@126.com (P.R.); mzj0712@hevttc.edu.cn (Z.M.); 2 Hebei Key Laboratory of Veterinary Preventive Medicine, College of Animal Science and Technology, Hebei Normal University of Science and Technology, Qinhuangdao 066000, China

**Keywords:** STTM-miR-5110, SOX10, MITF, melanocytes, melanogenesis

## Abstract

MicroRNAs (miRNAs) play important roles in the regulation of melanogenesis and coat color in mammals. Short tandem target mimics (STTMs) have been used to block the functions of small RNA in animals and plants. To investigate the role of miR-5110 in melanogenesis, STTM was used to block the expression of miR-5110 (STTM-miR-5110). Luciferase reporter assay data indicated the miR-5110 regulates *SOX10* expression by targeting its 3′-UTR. Overexpression of STTM-miR-5110 in alpaca melanocytes downregulated the expression of miR-5110 (decreased by about 38%, *p* < 0.05) and upregulated SOX10 mRNA (2.2-fold, *p* < 0.001) and protein (1.3-fold, *p* < 0.05) levels. Overexpression of STTM-miR-5110 in alpaca melanocytes increased the mRNA expression of melanogenic genes, including microphthalmia transcription factor (2.0-fold, *p* < 0.01), tyrosinase (1.6-fold, *p* < 0.01), tyrosinase-related protein 1 (approximately 3.9-fold, *p* < 0.001) and tyrosinase-related protein 2 (1.9-fold, *p* < 0.01). Overexpression of STTM-miR-5110 in alpaca melanocytes increased the protein expression of melanogenic genes, including microphthalmia transcription factor (1.9-fold, *p* < 0.05), tyrosinase (1.3-fold, *p* < 0.05), tyrosinase-related protein 1 (1.8-fold, *p* < 0.001) and tyrosinase-related protein 2 (1.6-fold, *p* < 0.05). The overexpression of pGL0-STTM-miR-5110 in alpaca melanocytes increased melanin production by approximately 26% (*p* < 0.05), pheomelanin production by approximately 38% (*p* < 0.05) and eumelanin production by approximately 56% (*p* < 0.001). In addition, overexpression of STTM-miR-5110 in alpaca melanocytes increased the TYR activity by 37% (*p* < 0.01). We also identified melanin granules in alpaca melanocytes transfected with STTM-miR-5110 under Fontana-Masson staining. These results suggest that STTM-miR-5110 upregulates melanogenesis by effectively blocking miR-5110 expression.

## 1. Introduction

Melanocytes are specialized cells found in the skin, derived from neural crest cells. They are located at the basal layer of the epidermic hair bulb, eyes, ears and meninges [1]. melanocytes have an important role in the skin’s innate immunity and determine skin color by producing melanin [2]. Melanin synthesis takes place in intracellular organelles named melanosomes [3]. Melanosomes are specialized, pigment-containing, lysosome-related organelles and can synthesize two types of melanin granules (eumelanin and pheomelanin), and the amount and distribution of two melanin types determines animal skin and coat color [2].

Alpaca (*Vicugna pacos*), a domestic mammal primarily specialized for fiber production [4] and exhibits over 22 natural coat colors, as a model to study the melanogenesis. Coat color formation of mammalias includes melanins production in melanocytes and melanins transferring from melanocytes to keratinocytes around [5], which is decided and regulated by many genes and microRNAs (miRNAs).

MicroRNAs (miRNAs) are a class of small, endogenous, noncoding RNAs approximately 22 nucleotides (nt) in length [6]. They induce post-transcriptional silencing of target sequences by interacting with their 3′-untranslated regions (3′-UTRs) of specific transcripts, leading to translational repression and gene silencing [7,8]. Many miRNAs regulate melanogenesis, which is responsible for coat color [9,10]. Previous research demonstrated that miR-5110 regulates pigmentation by co-targeting melanophilin and WNT family member 1 [4].

Short tandem target mimics (STTMs), which utilize target mimicry (TM) technology, are designed to inhibit the function of small RNA molecules in both animals and plants. STTMs are a powerful technology to complement existing small RNA sequestration [11]. STTMs are roughly 100 nucleotides in length and contain two miRNA-binding elements (such as two TMs in tandem) with a mismatch at the miRNA cleavage site spaced by a weak stem-loop linker of 48–88 nt [12]. STTMs induce the degradation of most, if not all, cognate miRNAs partly through the action of small degrading nucleases [11].

Bioinformatics analysis revealed sex-determining region Y-box 10 (*SOX10*) as another target gene of miR-5110. *SOX10* is an important transcriptional regulator in various cell types, including melanocytes [13]. Thus, we expected that the STTM of miR-5110 (STTM-miR-5110) would inhibit miR-5110′s role in upregulating melanogenesis in alpaca melanocytes by targeting *SOX10*. This study aimed to test this hypothesis.

## 2. Materials and Methods

### 2.1. Construction of Plasmids

STTM-miR-5110 was chemically synthesized as described previously [11]. The STTM-miR-5110 expression plasmid was constructed by inserting STTM-miR-5110 into the dual-luciferase vector pmirGL0 (Promega Corporation, Madison, WI, USA) to construct the expression plasmid pGL0-STTM-miR-5110. The null pGL0 vector was used as a negative control (NC). Luciferase reporter plasmids were constructed by cloning the 3′-UTR sequences of alpaca *SOX10* into the dual-luciferase pGL0 vector (Promega Corporation, Madison, WI, USA). Partial sequences of alpaca *SOX10* containing miR-5110 binding sites were amplified from alpaca skin cDNA via PCR using primers that included SacI (NEB, Ipswich, MA, USA) and XhoI (NEB, Ipswich, MA, USA) restriction sites. The PCR products and vector were digested with SacI and XhoI, then ligated to generate pGL0-SOX10-wt (wild type, WT) constructs. The miR-5110 binding sites in these plasmids were mutated using a Site-Directed Gene Mutagenesis Kit (Beyotime, Shanghai, China) according to the manufacturer’s instructions to obtain the pGL0-SOX10-mut plasmid. All constructs were confirmed by sequencing.

### 2.2. Cell Culture and Transfection

Melanocytes, grown to 77–80% confluent, were transfected with pGL0-STTM-miR-5110 and/or the pGL0 plasmid using Lipofectamine 2000 (Thermo Fisher Scientific, Waltham, MA, USA) following the manufacturer’s instructions. Three days after transfection, the melanocytes were collected for subsequent quantitative real-time PCR (qRT-PCR) and Western blotting analyses, respectively (*n* = 3).

### 2.3. Dual-Luciferase Assay for miRNA Target Validation

The HEK 293T cell line was obtained from National Collection of Authenticated Cell Cultures(Serial: SCSP-5209, Identifier: CSTR:19375.09.3101HUMSCSP5209). HEK 293T cells were cultured in DMEM containing 10% fetal bovine serum. For the luciferase reporter assay, 2 mg of the plasmid (including 4 groups: SOX10-wt and pGL0; SOX10-wt and pGL0-STTM-miR-5110; SOX10-mut and pGL0; SOX10-mut and pGL0-STTM-miR-5110) were co-transfected into HEK 293T cells using Lipofectamine 2000 (Thermo Fisher Scientific, Waitham, MA, USA). Luciferase activity was measured using the Dual-Luciferase Reporter Assay Kit (Promega Corporation). Firefly luciferase activity was normalized to Renilla luciferase activity to account for the transfection efficiency. Data are expressed as the mean ± standard deviation (SD; *n* = 3).

### 2.4. Quantitative Real-Time PCR (qRT-PCR) for miR-5110 and mRNA

Total RNA was extracted from melanocytes using TRIzol reagent (Thermo Fisher Scientific, Waltham, MA, USA) following the manufacturer’s instructions. For quantification of mRNA, 1 mg of total RNA was reverse-transcribed into cDNA with a TaKaRa Bio cDNA Synthesis Kit, following the manufacturer’s protocol. For quantification of miRNA, cDNA was generated using the cDNA Synthesis Kit (TaKaRa Bio, Dalian, China) with U6 reverse primer and a common primer, as previously described for real-time quantification of miRNAs [14]. qRT-PCR analyses for both experiments were conducted on a 7500 Fast Real-Time PCR System (Thermo Fisher Scientific, Waltham, MA, USA), employing SYBR Green PCR Master Mix (TaKaRa Bio, Dalian, China). The primer sequences are listed in Table 1. Three technical replicates were performed for each sample. The expression levels of miRNA and mRNA were calibrated against U6 small nuclear RNA and β-actin mRNA, respectively, with these endogenous controls serving as the reference genes for normalization. The relative abundance of miRNA and mRNA transcripts was assessed employing the comparative Ct (2^−ΔΔCt^) method via quantitative real-time PCR [15]. Data are expressed as the mean ± standard deviation (SD; *n* = 3).

### 2.5. Western Blotting Analysis

For Western blot analysis, the extracted protein samples from the melanocytes transfected with pGL0-STTM-miR-5110 and/or the pGL0 plasmid were performed employing sodium dodecyl sulfate-polyacrylamide gel electrophoresis (SDS-PAGE) with gel concentrations of 8% or 10%, prior to the electrophoretic transfer of the proteins to PVDF membranes. Blocking of the membranes was performed using 5% skim milk for 2 h. Subsequently, the membranes were probed with appropriately diluted primary antibodies directed against SOX10 (1:500), MITF (1:800), TYR(1:1000), TYRP1 (1:1000), TYRP2 (1:1000), and β-actin (1:1000), with incubation proceeding overnight at 4 °C. The membranes were washed four times using TBST, each wash lasting 5 min, and then incubated for 1 h at 37 °C with horseradish peroxidase-conjugated secondary antibody raised against rabbit IgG (1:10,000). Following four 5 min rinses with TBST, the antigen–antibody complexes were detected by enhanced chemiluminescence. Subsequent to imaging with a ChemiDOC XRS+ Imager (Bio-Rad, Hercules, CA, USA), a quantitative analysis of protein expression levels was performed based on the acquired immunoblot images utilizing Image-Pro Plus Software 6.0 (Olympus, Tokyo, Japan). Data are expressed as the mean ± standard deviation (SD; *n* = 3).

### 2.6. Immunocytochemistry

Following the transfection of melanocytes with the pGL0-STTM-miR-5110 or pGL0 plasmid, the cells were washed three times using PBS, each wash lasting 3 min, and were fixed using 4% paraformaldehyde. Subsequently, to block endogenous peroxidase activity, the cells were incubated at room temperature in 3% hydrogen peroxide for 15 min. After being washed using PBS, each wash lasting 5 min, the cellular samples were immersed in BSA and maintained at 37 °C for 25 min. Subsequently, the samples were finally incubated in a solution of the primary antibody (rabbit anti-SOX10) at 4 °C for a duration of overnight. After being washed three times using PBS, each lasting 5 min, the cells were incubated with horseradish peroxidase-conjugated anti-rabbit IgG for 30 min at 37 °C and observed by microscopy (Leica Microsystems, Buffalo Grove, IL, USA). IgG was used as the primary antibody against the NC.

### 2.7. Melanin Measurement

Following the transfection of melanocytes with the STTM-miR-5110 or pGL0 plasmid, the cells were harvested and rinsed with PBS. For the determination of total alkali melanin, the sample was mixed with 1 mL of 0.2 M NaOH, following which the absorbance was recorded at 475 nm using a spectrophotometer. For the determination of pheomelanin, the sample was solubilized in phosphate buffer (pH10.5) and cleared by centrifugation at 10,700× *g* for 10 min, following which the absorbance was recorded at 400 nm using a spectrophotometer. For the determination of eumelanin, the sample was hydrolyzed in hot 30% hypophosphoric acid and hydriodic acid solutions. After cooling, 50% ethanol was added and the sample was centrifuged at 2234× *g* for 10 min. Insoluble eumelanic pigments were selectively solubilized in hot sodium hydroxide and hydrogen peroxide, and the solution was cleared by centrifugation at 10,700× *g* for 1 min, following which the absorbance was recorded at 350 nm using a spectrophotometer. Melanin content was standardized against the total cell count. Each experiment included three biological replicates.

### 2.8. TYR Activity Measurement

Following the transfection of melanocytes with the STTM-miR-5110 or pGL0 plasmid, the cells were washed three times using PBS, each wash lasting 3 min. Subsequently, the cells were seeded into 96-well plates, with each well containing 100 μL of 1% Triton X-100, followed by 5 min of vortex mixing. After incubation at room temperature for one hour, 50 μL of 1% Levodopa was introduced into each well, and the plates were further incubated at 37 °C for 2 h. Tyrosinase (TYR) activity was assessed spectrophotometrically based on absorbance measurements at a wavelength of 460 nm.

### 2.9. Fontana-Masson Staining

Following the transfection of melanocytes with the STTM-miR-5110 or pGL0 plasmid, the cells were washed using PBS (pH 7.4) and fixed in 4% formaldehyde for 20 min. Cell staining was performed by incubation with an ammoniacal silver solution (Fontana-Masson kit, Abcam, UK) at 55 °C in the dark for 1 h. Subsequently, the stained cells were washed multiple times with distilled water to ensure complete removal of unbound stain. Then, the cells were subjected to a 2 min incubation in a solution containing 0.2% gold chloride and 5% sodium thiosulfate, and were subsequently counterstained with nuclear fast red for 5 min. After each staining step, a distilled water wash was performed, neutral balsam was used to cover the slides, and melanin was evaluated under a light microscope (Leica Microsystems, Buffalo Grove, IL, USA).

### 2.10. Statistical Analysis

All data were analyzed using T-tests, with normality test assessed by the Shapiro–Wilk test. This analysis quantified the expression levels of target miRNA and mRNA, the corresponding protein abundance, relative luciferase activities, and intracellular melanin concentrations, with all computations performed using GraphPad Prism (version 9.0). Data are reported as means ± SD. Values of *p* < 0.05 were significant.

## 3. Results

### 3.1. STTM-miR-5110 Reduces the Levels of miR-5110 in Alpaca Melanocytes

**Figure 1:**
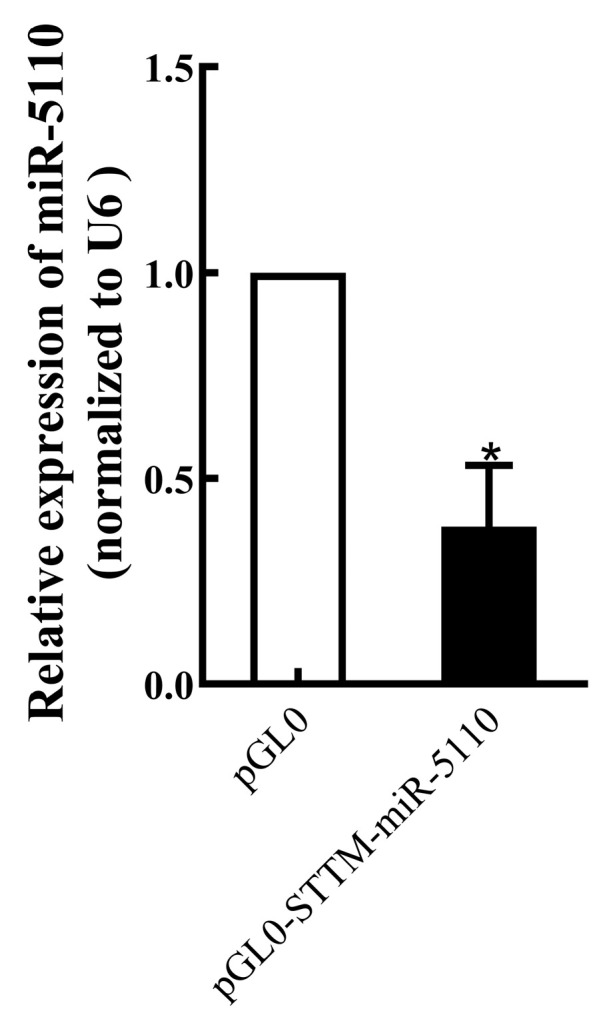
Real-time PCR analysis of miR-5110 expression in melanocytes transfected with the STTM-miR-5110 expression plasmid. Data are expressed as mean ± SD (*n* = 3). * *p* < 0.05.

The expression level of miR-5110 in melanocytes transfected with STTM-miR-5110 was assessed. Expression of miR-5110 in melanocytes transfected with STTM-miR-5110 was significantly lower (by about 38%, *p* < 0.05) than in those transfected with the PGL0 plasmid (Figure 1).

### 3.2. STTM-miR-5110 Targeting SOX10

**Figure 2:**
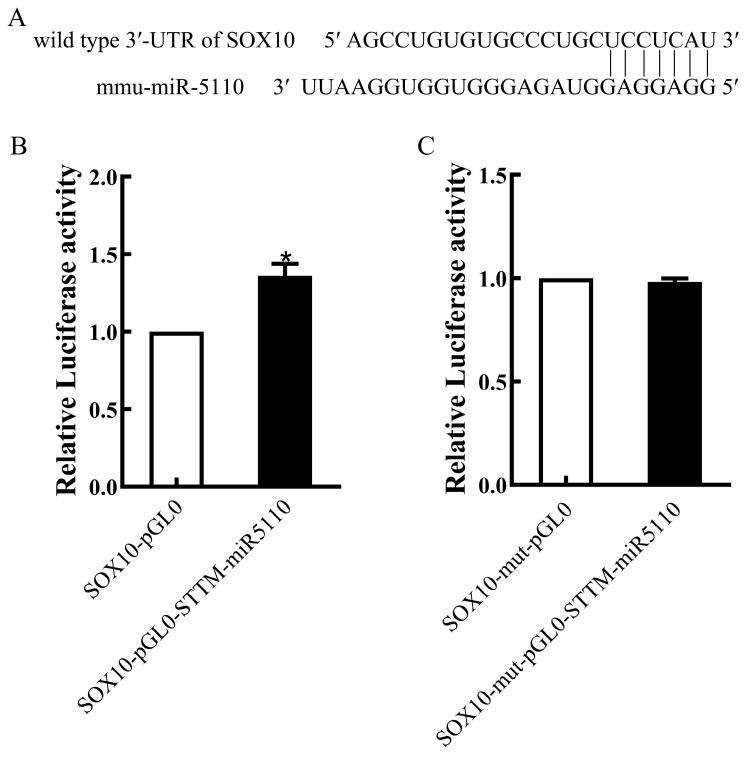
miR-5110 targeting 3′-UTR of *SOX10*. (**A**) Predicted miR-5110 binding site in the 3′-UTR of *SOX10*. (**B**,**C**) Luciferase reporter assays in 293T cells co-transfected with the wild-type 3′-UTR of *SOX10* and the STTM-miR-5110 or the pGL0 plasmids. The data from dual luciferase assays are expressed as the mean relative luciferase activities ± SD (*n* = 3). * *p* < 0.05.

Bioinformatics analysis using the miRBase software (version 22.0) revealed that *SOX10* is a potential target gene of miR-5110 (Figure 2A). To confirm that STTM-miR-5110 bound to the 3ʹ-UTR of *SOX10*, we constructed plasmids containing the WT or mutant 3ʹ-UTR of *SOX10*. The luciferase activity of cells transfected with the pGL0-STTM-miR-5110 and pGL0-SOX10 was increased 1.36-fold relative to that of cells co-transfected with the pGL0 and pGL0-SOX10 (*p* < 0.05) (Figure 2B). The luciferase activity of cells transfected with the pGL0-STTM-miR-5110 and pGL0-SOX10-mut was similar to the reporter activity in cells co-transfected with pGL0 and pGL0-SOX10-mut (Figure 2C). These data demonstrated that STTM-miR-5110 significantly increased the luciferase activity by targeting the *SOX10* 3ʹ-UTR.

### 3.3. Effect of STTM-miR-5110 Overexpression on mRNA and Protein Levels of SOX10

**Figure 3:**
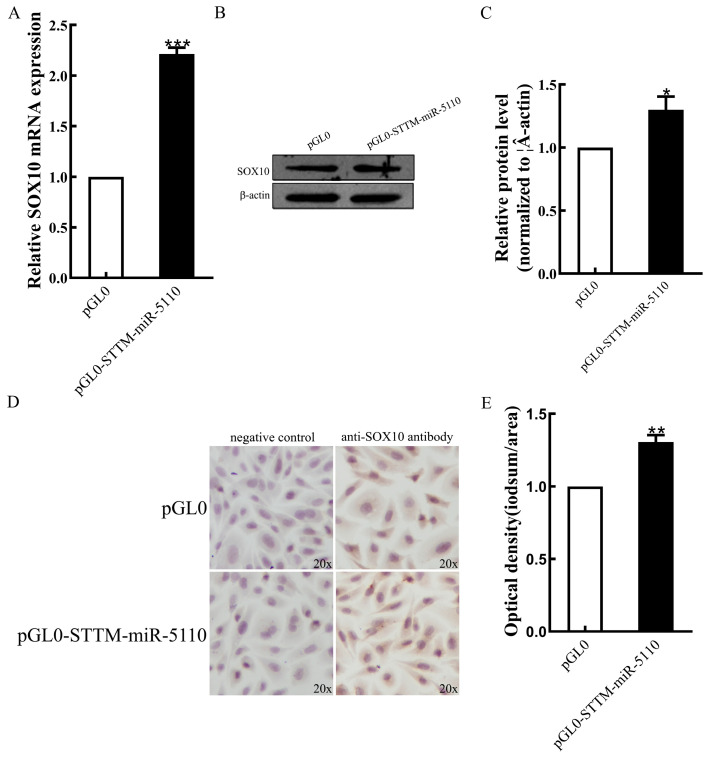
Effects of STTM-miR-5110 on the expression levels of SOX10. (**A**) The mRNA expression level of *SOX10* in melanocytes of alpaca transfected with STTM-miR-5110 expression plasmids. Analysis of SOX10 protein expression in melanocytes of alpaca transfected with STTM-miR-5110 expression plasmids by Western blot detection (**B**,**C**) and immunocytochemistry (**D**,**E**), respectively. The data are expressed as the mean ± SD (*n* = 3). * *p* < 0.05, ** *p* < 0.01, *** *p* < 0.001.

Overexpression of STTM-miR-5110 resulted in an increase in the levels of both SOX10 mRNA (2.2-fold, *p* < 0.001) (Figure 3A) and SOX10 protein (1.3-fold, *p* < 0.05) (Figure 3B,C and Figure S1), as analyzed by qRT-PCR and Western blotting. Immunohistochemical analysis of SOX10 expression in the cytoplasm of alpaca melanocytes, optical density of cells transfected with the pGL0-STTM-miR-5110 was increased 1.3-fold relative to that of cells co-transfected with the pGL0 (*p* < 0.01) (Figure 3D,E, Table S1). These data indicated that *SOX10* is regulated by miR-5110 through STTM.

### 3.4. Effect of STTM-miR-5110 Overexpression on the Expression of Melanogenic Genes

**Figure 4:**
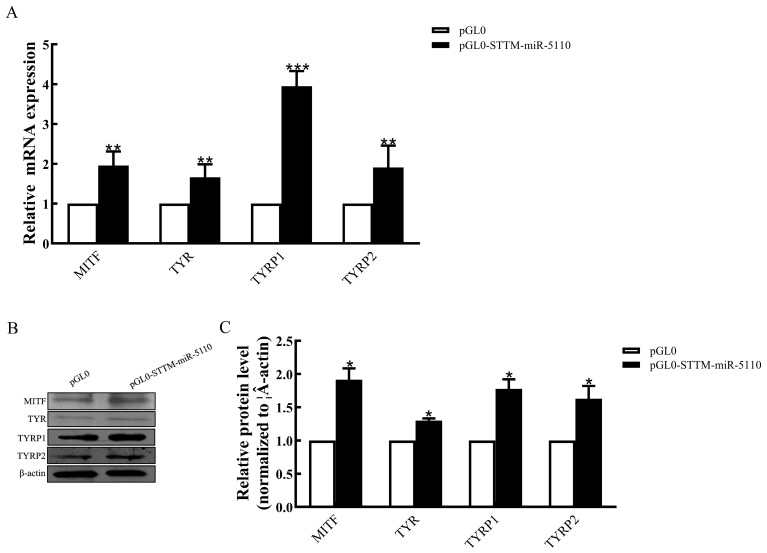
Effects of STTM-miR-5110 on the expression of the melanogenic genes in melanocytes. (**A**) mRNA expression of the MITF, TYR, TYRP1, and TYRP2 genes in melanocytes of alpaca transfected with the STTM-miR-5110 expression plasmid. (**B**,**C**) Analysis of MITF, TYR, TYRP1, and TYRP2 protein expression in melanocytes transfected with the STTM-miR-5110 expression plasmids. The data were normalized to β-actin levels and are expressed as the relative fold change. The data are expressed as the mean ± SD (*n* = 3). * *p* < 0.05; ** *p* < 0.01; *** *p* < 0.001.

To assess the functional role of STTM-miR-5110 in melanogenesis within alpaca melanocytes, we analyzed the transcriptional activity of key genes associated with this pathway. Overexpression of STTM-miR-5110 in alpaca melanocytes increased the mRNA expression of melanogenic genes, including microphthalmia transcription factor (2.0-fold, *p* < 0.01), tyrosinase (1.6-fold, *p* < 0.01), tyrosinase-related protein 1 (3.9-fold, *p* < 0.001) and tyrosinase-related protein 2 (1.9-fold, *p* < 0.01) (Figure 4A). Overexpression of STTM-miR-5110 in alpaca melanocytes increased the protein expression of melanogenic genes, including microphthalmia transcription factor (1.9-fold, *p* < 0.05), tyrosinase (1.3-fold, *p* < 0.05), tyrosinase-related protein 1 (1.8-fold, *p* < 0.001) and tyrosinase-related protein 2 (1.6-fold, *p* < 0.05) (Figure 4B,C and Figure S2).

### 3.5. Effect of STTM-miR-5110 Overexpression on Melanin Production and TYR Activity

**Figure 5:**
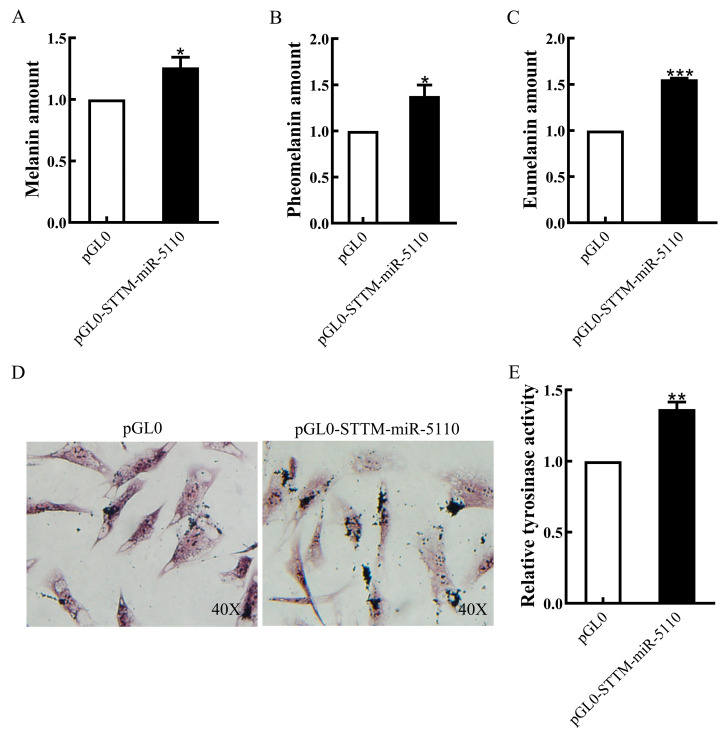
Effects of STTM-miR-5110 overexpression on melanin production and TYR activity. The amount of melanin (**A**), pheomelanin (**B**), and eumelanin (**C**) in STTM-miR-5110 overexpressing melanocytes of alpaca. (**D**) Melanin distribution in melanocytes overexpressing pGL0-STTM-miR-5110 and pGL0 using Fontana-Masson staining. (**E**) The TYR activity in STTM-miR-5110 overexpressing melanocytes of alpaca. The data are expressed as the mean ± SD (*n* = 3). * *p* < 0.05; ** *p* < 0.01; *** *p* < 0.001.

To investigate the function of STTM-miR-5110 in melanin production, we quantified the production of total alkali melanin, eumelanin, and pheomelanin in alpaca melanocytes overexpressing STTM-miR-5110. The results demonstrated that the overexpression of pGL0-STTM-miR-5110 in alpaca melanocytes increased melanin production by approximately 26% (*p* < 0.05, Figure 5A), pheomelanin production by approximately 38% (*p* < 0.05, Figure 5B) and eumelanin production by approximately 56% (*p* < 0.001, Figure 5C). To confirm the localization of melanin in melanocytes overexpressing STTM-miR-5110 by the Fontana-Masson staining. The content and distribution of melanin in melanocytes overexpressing STTM-miR-5110 was significantly higher than that of cells transfected with the pGL0 plasmid (Figure 5D). To investigate whether STTM-miR-5110 affects TYR activity, we measured the activity of alpaca melanocytes overexpressing STTM-miR-5110. The results showed that the overexpression of pGL0-STTM-miR-5110 in alpaca melanocytes increased TYR activity by 37% (*p* < 0.01, Figure 5E).

## 4. Discussion

In animal models, STTM constructs localized within intronic regions effectively suppress endogenous miRNA activity, leading to the depression of a luciferase reporter gene fused to the 3′-UTR of miRNA target genes [11]. Consistent with this mechanism, our luciferase reporter assay confirmed that miR-5110 directly targets 3′-UTR of *SOX10* (Figure 2). Furthermore, transfection of alpaca melanocytes with STTM-miR-5110 indeed led to a significant knockdown of miR-5110 levels (Figure 1), which established the foundation for the subsequent phenotypic and mechanistic analysis.

Melanocytes, residing in the skin, hair, and ocular choroid, are responsible for the synthesis of melanin pigments. These specialized cells produce two types of melanin: eumelanin (brown-black) and pheomelanin (yellow-red) [16]. Several microRNAs (miRNAs) implicated in the regulation of melanogenesis have been identified across different animal species. In a previous study, we showed that miR-5110 regulates pigmentation by co-targeting *MLPH* and *WNT1* [4]. In the present study, we identified a novel target gene, *SOX10*, and elucidated its downstream pathway. Our central finding is that knockdown of miR-5110 via STTM significantly increased melanin, pheomelanin and eumelanin production in alpaca melanocytes (Figure 5A–C).

SRY HMG box (SOX) proteins are transcription factors that belong to the HMG box superfamily of DNA-binding proteins. These proteins play key roles during development. *SOX10* belongs to the SOX-E group, which also includes *SOX8* and *SOX9* [17]. As a key transcriptional regulator, *SOX10* plays a pivotal role in the formation of neural crest cells across a wide range of vertebrate species, including rodents [18], zebrafish [19], Xenopus [20], and humans [21]. *SOX10* plays a pivotal role in the initial specification of the melanocyte lineage and is essential for the survival of migrating neural crest cells [22]. Studies in Xenopus have demonstrated that *SOX10* plays a critical role in melanocyte development, as its targeted overexpression directly promotes the expansion of the melanocyte population [20]. To elucidate the mechanism behind the increased melanogenesis, we focused on the SOX10-MITF axis. Since *SOX10* is a direct target of miR-5110 (Figure 2), its expression was expected to be derepressed upon miR-5110 knockdown. Indeed, our qRT-PCR and Western blot analysis confirmed that both SOX10 mRNA and protein levels were significantly upregulated in STTM-miR-5110-transfected cells compared to pGL0 (Figure 3). Accordingly, we observed a concomitant increase in the expression of MITF at both transcriptional and translational levels in our experiment. *SOX10* is a pivotal transcriptional regulator of *MITF* [23,24,25]. The melanocyte-specific transcription factor MITF-M functions as a central regulator by modulating the expression of key melanogenic enzymes, such as tyrosinase (*TYR*), tyrosinase-related protein 1 (*TYRP1*), and dopachrome tautomerase (*DCT/TYRP2*) [26].Consistent with this established hierarchy, the upregulation of MITF in melanocytes was followed by a marked induction of TYR, TYRP1 and DCT/TYRP2 expression. Tyrosinase, a key enzyme in melanin synthesis, is encoded by the *TYR* gene situated on chromosome 7 in the mouse genome [27]. Tyrosinase is the rate-limiting enzyme responsible for melanin synthesis. This enzyme mediates the oxidation of l-dihydroxy-phenylalanine (DOPA) to DOPA quinone. The resultant quinone serves as a common biosynthetic precursor for the two principal forms of melanin: eumelanin and pheomelanin [28].

In addition, *DCT/TYRP2* is a direct target gene of *SOX10* in melanocytes [13]. *DCT/TYRP2* expression was previously shown to be controlled by the *MITF* transcription factor [29]. TYR is required for eumelanin and pheomelanin synthesis, whereas *TYRP1* and *TYRP2* are crucial for eumelanin synthesis [30]. As expected, eumelanin and pheomelanin production was increased by STTM-miR-5110 overexpression.

The promising data presented in this study should be interpreted in light of a key methodological constraint: all evaluations were conducted in melanoctyes model rather than in the target species, alpaca. This experimental design reflects the consensus that melanocytes offer a standardized and controllable platform for the function of STTM in melangenesis. Moreover, this approach aligns with ethical frameworks for exploratory investigation and remains logistically practical before advancing to alpaca studies.

Future research should aim to develop and utilize an alpaca model to directly evaluate whether the knockdown of miR-5110, mediated by STTM technology, can effectively modulate coat color phenotypes. This would provide definitive evidence for its potential application in biological research or animal biotechnology, bridging the gap between cellular mechanisms and organismal traits. Furthermore, considering biosafety, we must maintain a cautious attitude when extrapolating this conclusion to other species.

## 5. Conclusions

STTM-miR-5110 is an artificial biological tool that controls melanogenesis by decreasing the expression of miR-5110. This process regulates the levels of SOX10, and in turn, affects melanin secretion in melanocytes.

## Figures and Tables

**Table 1:** miR-5110 and mRNAs primer sequences.

Primer Name	Primer Sequence 5′–3′	Application
SOX10-wt	F: GCGAGCTCTCACCACCAGTGCCCACA	Luciferase reporter-wt
	R: GCCTCGAGGTCCCACCCTGCTCTTTAC	
SOX10-mut	F: GAAGCTGTTGTACGAATTGATGATGAACAAAAGTCATCTGT	Luciferase reporter-mut
	R: ACAGATGACTTTTGTTCATCATCTTAACGTACAACAGCTTC	
miR-5110	F: ACACTCCAGCTGGGGGAGGAGGTAGAGGGTGGT	Real time PCR
	R: TGGTGTCGTGGAGTCG	
Common-R	CGAGCAGTGCAGGGTCCGAGGT	RT-PCR
U6	F: CTCGCTTCGGCAGCACA	Real time PCR
	R:GTCGTATCCAGTGCAGGGTCCGAGGTATTCGCACTGGATACGACTCATCT	
SOX10	F: AAGCCTCACATCGACTTCGG	Real time PCR
	R: GGTCAGAGATGGCCGTGTAG	
MITF	F: TCCCAAGTCAAATGATCCAG	Real time PCR
	R: GAGCCTGCATTTCAAGTTCC	
TYR	F: GCTTTAGCAACTTCATGGGA	Real time PCR
	R: CTTGTTCTTCTCTGGGACAC	
TYRP1	F: GCCTTCTTTCTCCCTTC	Real time PCR
	R: CAGACCACTCGCCATT	
TYRP2	F: AGCAGACGGAACACTGGACT	Real time PCR
	R: GCATCTGTGGAAGGGTTGTT	
β-actin	F: CTAAGGAGAAGGGCCAGTCC	Real time PCR
	R: CTCAAGTTGGGGGACAAAAA	

F: forward primer; R: reverse primer.

## Data Availability

The raw data supporting the conclusions of this article will be made available by the authors on request.

## Supplementary Materials

The following supporting information can be downloaded at: https://www.mdpi.com/article/10.3390/cimb48010072/s1.
