# Supplementary material for: Effect of Short Tandem Target Mimic miR-5110 on Melanogenesis in Melanocytes of Alpaca (Vicugna pacos)
Source: Curr Issues Mol Biol. 2026 Jan 10;48(1):72. doi: 10.3390/cimb48010072 (PMC12839781; doi:10.3390/cimb48010072)
Supplement: Supplementary file 1 [file cimb-48-00072-s001.zip › cimb-4067019-supplementary/Figures= S1 and Figure. S2.pdf]

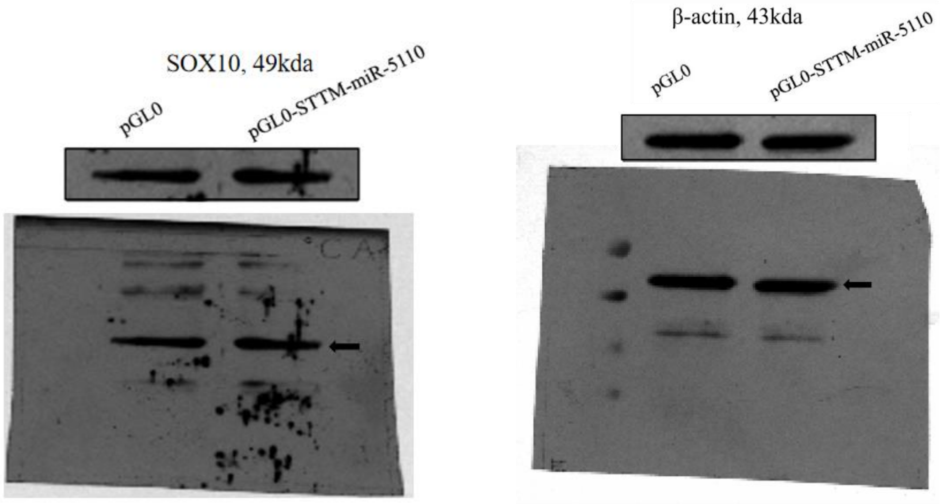

Figure S1. Analysis of SOX10 protein expression in melanocytes of alpaca transfected with STTM-miR-5110 expression plasmids by Western blot detection.

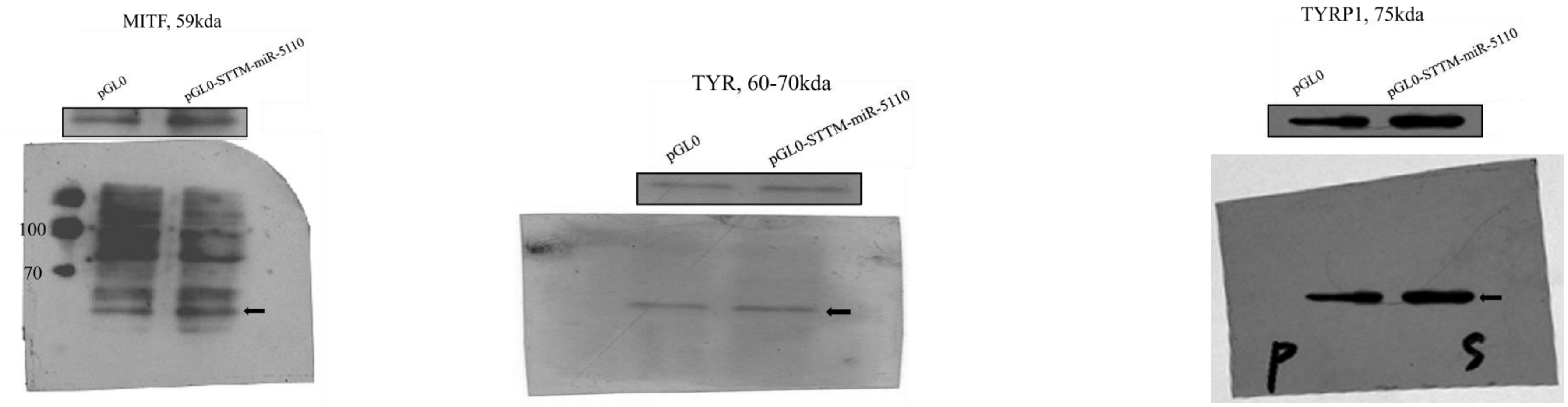

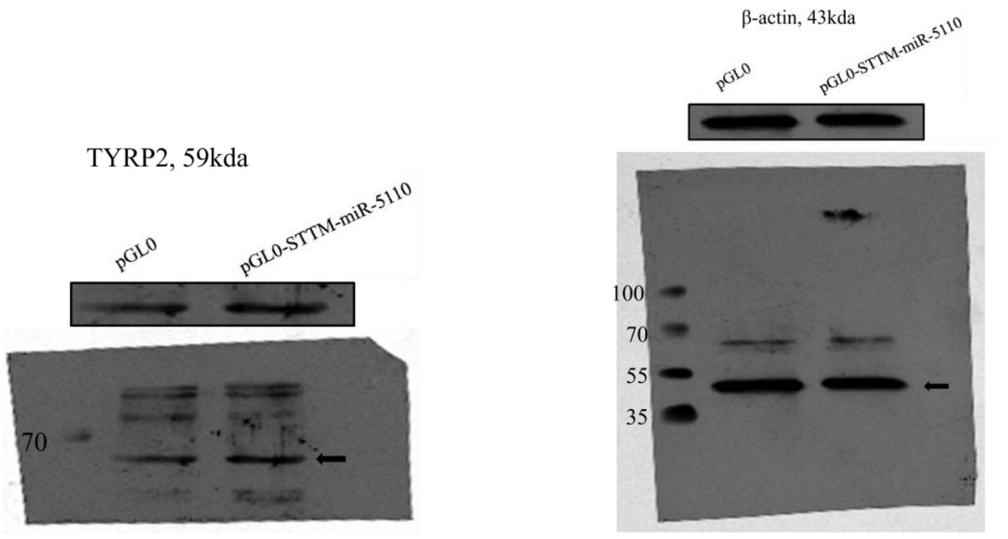

Figure S2. Analysis of MITF, TYR, TYRP1, and TYRP2 protein expression in melanocytes transfected with the STTM-miR-5110 expression plasmids by Western blot detection.
